# Medical Education Meets Modern Pedagogy: Medical Students Perspective

**DOI:** 10.31729/jnma.9128

**Published:** 2025-06-30

**Authors:** Sandip Bhusal, Aashis Poudel

**Affiliations:** 1 Patan Academy of Health Sciences, Lagankhel, Lalitpur, Nepal

**Keywords:** *medical education*, *medical student*, *pedagogy*, *problem-based learning*

## Abstract

The principles of adult learning have significantly transformed medical education, moving away from traditional lecture-based methods to a more interactive, self-directed, and problem-centered approach. This article reflects on the personal experiences of medical students steering into adult learning. Problem-Based Learning, clinical presentations, feedback methods, and innovative assessment tools were the components of this pedagogy. This helped foster critical skills such as communication, teamwork, and self-reflection. This perspective emphasizes the importance of adopting adult learning methodologies to enhance the quality of education and patient care.

## INTRODUCTION

The teaching strategies in the past were mainly teacher- centered and lecture-based with non integrated learning.^1^ Adult learning which dates back to 1920s, is a pedagogical approach grounded in the principles of adult education theory.^2^ It recognizes that adult learners exhibit distinct characteristics and learning preferences and is characterized by self-directedness, a readiness to learn based on life experiences, a problem-centered approach, intrinsic motivation, and a focus on practical applicability.^3^ Though lectures are still the mainstay for delivering academic knowledge in majority of medical schools, the incorporation of adult learning principles into our medical school curriculum can maximize the effectiveness of teaching-learning process.^4^ It can help to foster skills like communication, leadership, critical thinking, patient centered care, and problem solving approaches.^5^

## EXPERIENCE

Transitioning from high school to medical school was overwhelming given the extensive syllabus and rigorous expectations. We were wondering how this heavy content could be delivered in such a small-time frame and how we could retain all these information. Teaching at our medical school emphasized selfdirected learning, a core component of adult learning. So, to familiarize this technique, we underwent a two- month "Introductory Block" before commencing basic sciences.

One of the aspects we learned was providing effective feedback. Initially, we believed feedback only addressed shortcomings. However, we practiced the "Sandwich" method, where positive comments bookend constructive criticism.^6^ One group presented, while another gave feedback using this method. Reinforcing specific, timely feedback helped refine our performance. We also practiced PowerPoint presentations, learning the significance of body language, tone, facial expressions, and engagement strategies such as two-way communication and interactive sessions.

We developed scientific writing skills, learning to extract and summarize information, take references from articles, and avoid plagiarism. Techniques like highlighting key points, using diagrams, and creating flowcharts helped us retain the information. We focused on evidence-based resources like PubMed, UpToDate, and HINARI, ensuring credibility. We felt joy as we started discussing articles from Pubmed with proper references.

We realized that active involvement in learning and peer teaching proved more effective than passive lectures. Our curriculum incorporated Problem-Based Learning (PBL), clinical presentations, self-study, and diverse assessment tools to foster active engagement, critical thinking, and self-directed learning for mastering both basics and clinical sciences.

## PROBLEM-BASED LEARNING

PBL is based on self-directed learning. It is an instructional approach that is student centered. Trulls et al. claimed that PBL methodology was more successful than traditional lecture-based methods in improving communication, problem-solving, and selfdirected learning skills.^7^

Patan Academy of Health Sciences adopted PBL as the primary pedagogical strategy to nurture self-directed learning, leadership, communication, and reflective thinking. Small groups of 6-10 students, guided by a faculty tutor, had three PBL sessions weekly, with wrap-up sessions on Fridays. We adhered to the ground rules for discussion ensuring respect and equal participation.

Each week, we analyzed a clinical case, having triggers such as include clinical information, investigation data, or images of the case. These cases were collaboratively designed by faculty members from various departments to ensure comprehensive coverage of the learning objectives across the blocks. The entire educational process was student-directed. To keep the discussion on track, our tutor used to steer the conversation whenever required to keep the discussion on track. This helped to ensure that the PBL objectives were met. We engaged in critical analysis, hypothesized the problems, presented solutions, arguments and counterarguments.

In subsequent sessions, we discussed learning issues from the previous sessions. Then our tutor provided new triggers. We had the week learning objectives by the end of final session. In addition to fulfilling these objectives, the discussions allowed us to explore broader clinical issues. In the wrap-up session each group presented the week’s objectives via Powerpoint Presentation. This was followed by a question-and-answer session with faculties. We asked the issues that were not solved by the group to content expert after the presentation. This helped us clarify complex concepts, gain deeper insights from the content expert. In course of time, we learned how we could help those students who were not doing well, either by reinforcing them to speak or creating a nice environment where everyone feels good to share their opinion. PBL enhanced our knowledge, presentation skills, and ability to seek expert guidance. Our experience aligns with findings from a study by Arienti et al. which found that after a structured PBL intervention, students showed statistically significant gains in evidence based practice domains, including terminology (from 54% to 65%) and practice (from 41% to 55%), at p < 0.001.^8^

## CLINICAL PRESENTATION

We engaged in case-based discussions using paper cases, fostering critical thinking and peer learning within our small groups. Our faculty members acted as facilitators, guiding us in the learning process rather than delivering lectures. We dissected various topics during discussions, ensuring clinical correlations with theoretical knowledge. Faculty members encouraged us to determine which investigations to order based on clinical presentation, making us realize that history and examinations alone can rule out many diagnoses.

This learning methodology empowered us to take responsibility for our education and apply theoretical knowledge to practical scenarios. Observing similar cases in the emergency room and outpatient departments solidified our understanding and boosted our confidence in making differential diagnoses. After forming a list of differentials, we conducted investigations to confirm or rule out those differentials. Our mentors provided patient management drills, further enhancing our diagnostic and management skills.

## SELF-STUDY

We initially found it surprising that Wednesdays were entirely dedicated to self-study. Additional self-study slots were also incorporated throughout the week schedule. We used this time to explore learning issues related to our PBL cases, engage in research activities, prepare presentations for clinical presentations, and spend hours studying in the library.

Having structured self-study time allowed us to identify personalized learning strategies and complete the syllabus at our own pace without undue pressure. By fostering autonomy, these sessions helped us develop effective learning habits and achieve a healthy work- life balance.

## ASSESSMENT TOOLS

**Assessment Tools:**

Our medical school introduced a unique and comprehensive evaluation system emphasizing both formative and summative assessments. One notable method was process evaluation that assessed our performance in Problem-Based Learning (PBL) sessions in basic sciences. Faculty members used the Tutor Assessment of Student (TAS) tools to evaluate our self-directed learning, communication, leadership, and reflectiveness, assigning scores-0 for poor, 1 for needs improvement and 2 for good performance. We were motivated to work on our weakness and further strengthen our strengths in further PBL sessions. It was exciting and fruitful to hear about our strengths from different tutors, to know our areas of improvement and on how we were improving on those aspects.

In clinical sciences, process evaluation encompassed learning logs, Mini Clinical Evaluation Exercises (mini- CEX), Directly Observed Procedural Skills (DOPS), and TAS evaluations. During clinical rotations, we were assigned individual mentors. Each week, we followed a patient case from admission to discharge, documenting the patient's history, physical examination findings and management.

We had to maintain learning logs weekly which contained three sections. In Section A focused on patient demographics, history, physical examination findings, investigations, and management. In Section B, emphasized the relevant etiopathogenesis and evidence-based treatment. Section C encouraged selfreflection, prompting us to analyse our interactions with the patient, evaluate the presentation, and identify key learning points. We presented our logs to our mentors, did in-depth discussion on the case bridging our theoretical knowledge with practical application, enhancing understanding and feedback on how we could improve.

During mini-CEX assessments, we performed brief history-taking and detailed physical examinations under supervision of our mentor. For DOPS, we carried out procedural tasks such as catheterization, intravenous cannulation, venipuncture, and electrocardiogram interpretation. Mentors supervised us, provided constructive feedback, and recorded our performance for evaluation.

In addition to process evaluations, we completed traditional assessments, including multiple-choice questions, problem-based questions, Objective Structured Clinical Examinations/Objective Structured Practical Examinations, clinical reasoning assessments, and viva voce exams. The diverse assessment methods helped us refine clinical skills, promote structured learning, and facilitate continuous self-improvement.

## WAY FORWARD

Recognizing medical students as adults is essential because it emphasizes their self-directedness, motivation, and capacity to engage actively in their education. In the dynamic and rapidly evolving field of medicine, the traditional approach can be inadequate for preparing the young minds of future doctors to meet the complex and multifaceted needs of patients. This gap can be filled by adult learning methodologies. Incorporation of adult learning makes medical professionals not just knowledgeable, but also helps to foster the skills like leadership, communication skills, mass speaking, critical thinking, reflectiveness, and lifelong learning. This ultimately enhances the quality of care and making a substantial difference in the lives of the patients.
